# Integrated proteomics and single-cell transcriptomics reveal potential therapeutic targets in Wilson’s disease patients

**DOI:** 10.3389/fimmu.2025.1635137

**Published:** 2025-10-01

**Authors:** Yue Qi, Minghui Sun, Fang Xu, Hao Zhou, Lihua Yuan, Xinlei Yu, Sirui Cao, Rui Hua

**Affiliations:** Department of Hepatology, Center of Infectious Diseases and Pathogen Biology, The First Hospital of Jilin University, Changchun, Jilin, China

**Keywords:** Wilson’s disease, proteomics, single-cell transcriptomics, ITIH1, TTR, PI3K-Akt pathway

## Abstract

**Purpose:**

Wilson’s Disease (WD), an autosomal recessive *ATP7B* mutations disorder causing copper accumulation, poses diagnostic challenges. This study used proteomics and single-cell transcriptomics to identify WD mechanisms and therapeutic targets.

**Methods:**

Proteomic analysis was conducted on clinical samples from WD patients and the control group, followed by validation via ELISA. Subsequently, an integrated analysis was conducted by combining these data with single-cell RNA sequencing data from the database. Analytical content included differential expression, functional enrichment, drug target prediction, immune infiltration, and subtype-specific biomarker screening via LASSO/SVM-REF.

**Results:**

Proteomic analysis identified 420 differentially expressed proteins (266 upregulated, 154 downregulated) in WD patients compared with healthy controls, with significant enrichment in inflammatory pathways. Integration with DrugBank revealed eight hub proteins with high diagnostic accuracy (AUC > 0.9), among which Inter-alpha-trypsin inhibitor heavy chain 1 (ITIH1) and Transthyretin (TTR) may regulate the PI3K-Akt signaling pathway. Subsequently, ELISA validation confirmed significantly reduced levels of TTR, Ceruloplasmin (CP), and ITIH1 proteins in WD. Considering the heterogeneity of the WD microenvironment and single-cell diversity, further single-cell transcriptomic analysis was performed. The results revealed immune dysregulation, characterized by increased macrophage infiltration and reduced T/NK cell proportions, and PI3K-Akt-mTOR pathway enrichment in macrophages. For subtype-specific analysis, six key proteins were identified to distinguish hepatic and brain subtypes (AUC > 0.9).

**Conclusions:**

The hub proteins and subtype-specific biomarkers identified in this study provide potential targets for the precise treatment of WD, while emphasizing the critical role of the PI3K-Akt pathway in WD.

## Introduction

Wilson’s disease (WD), also known as hepatolenticular degeneration, is a rare autosomal recessive disorder caused by mutations in the adenosine triphosphatase copper transporting beta (*ATP7B*) gene, which encodes a copper-transporting ATPase (ATPase 7B) cooperating with the antioxidant protein 1 (ATOX1) for hepatic copper excretion (1). Impaired function of *ATP7B* leads to pathological copper accumulation primarily in hepatocytes, which released the excess copper into bloodstream, with very toxic form affecting other organs, including the brain, kidneys, and cornea (2). Hepatic involvement ranges from asymptomatic elevation of liver enzymes to acute liver failure or cirrhosis (3). Brain symptoms, including dystonia, tremors, and psychiatric disturbances, typically manifest in the second or third decade of life (4). Additionally, patients may present with Kayser-Fleischer (K-F) rings, renal tubular acidosis, or hemolytic anemia (5–7). Clinically, WD is classified into hepatic, brain, and mixed subtypes, each with distinct pathological features and progression patterns (8). Despite its well-characterized genetic basis, WD remains challenging to diagnose early due to its phenotypic variability and nonspecific initial symptoms. Delayed diagnosis often results in irreversible organ damage, underscoring the urgent need for biomarkers that can facilitate early intervention and improve patient outcomes (9, 10).

Current treatments, such as copper chelators (e.g., penicillamine) and zinc salts, aim to reduce copper overload but are associated with significant side effects and variable efficacy (11, 12). Moreover, a subset of patients develops end-stage liver disease or severe brain disability despite therapy (13), highlighting gaps in understanding the molecular mechanisms driving WD pathogenesis. Identifying novel therapeutic targets, particularly those involved in copper toxicity or inflammatory cascades, could pave the way for more effective and personalized treatments (14, 15).

Recent advances in high-throughput technologies, particularly proteomics and single-cell transcriptomics, have revolutionized the study of complex diseases like WD (16). Proteomics enables systematic quantification of protein expression, post-translational modifications, and interactions, offering insights into disease-associated pathways and potential drug targets. For instance, large-scale proteomic studies have identified dysregulated proteins in WD patients, such as ceruloplasmin (CP) (17), which is implicated in copper transport and inflammation (18). However, bulk proteomics lacks cellular resolution, masking heterogeneity within immune microenvironment affected by WD.

Single-cell RNA sequencing (scRNA-seq) addresses this limitation by profiling gene expression at the individual cell level, revealing cell-type-specific responses to copper toxicity and immune microenvironment remodeling. In WD, scRNA-seq is expected to elucidate how copper accumulation differentially impacts hepatocytes, astrocytes, or immune cells, thereby uncovering subtype-specific mechanisms. For example, recent studies in other metabolic liver diseases have demonstrated the power of single-cell approaches to identify rare cell populations driving fibrosis or inflammation (17, 19, 20). Integrating proteomics with scRNA-seq provides a complementary perspective, linking protein-level alterations to transcriptional changes and cellular dynamics.

This study leverages both technologies to dissect the molecular landscape of WD. By combining proteomic profiling of patient plasma with single-cell transcriptomics of the hepatic microenvironment, we aim to: 1) identify dysregulated proteins and pathways contributing to WD progression; 2) characterize immune cell heterogeneity and its role in copper-induced tissue injury; 3) and discover subtype-specific biomarkers with diagnostic and therapeutic potential. By combining macro-level pathway analysis with micro-level cellular insights, this approach seeks to uncover novel mechanisms of copper toxicity and immune dysregulation, offering translational opportunities for precision diagnosis and therapy in WD.

## Materials and methods

### Sample cohort and data collection

#### Study participants

A total of 30 samples were included in the proteomic analysis, among which 20 were WD patients with a male-to-female ratio of 9: 11, consisting of 8 cases of hepatic subtype (HS), 4 cases of brain subtype (BS), and 8 cases of mixed subtype (MS); the remaining 10 were healthy controls (male: female = 5: 5). For Enzyme-Linked Immunosorbent Assay (ELISA) validation, 10 WD patients and 10 healthy controls were enrolled, with a male-to-female ratio of 5: 5 in both groups. All samples were obtained from the Department of Biobank, Division of Clinical Research, The First Hospital of Jilin University. Diagnosis of WD was confirmed by *ATP7B* genetic sequencing, CP reduces the increase in 24-hour urinary copper, and clinical evaluation, including the presence of K-F rings, brain symptoms. Exclusion criteria included concurrent viral hepatitis, autoimmune liver disease, alcoholic liver disease, drug-induced liver disease, and simple fatty liver. In addition, WD disease is classified into three types clinically: 1) the hepatic type involves hepatitis, cirrhosis, and liver function impairment, 2) the brain type manifests with brain symptoms such as Parkinson’s syndrome and movement disorders and 3) the mixed type exhibits characteristics of both types. Healthy controls were individuals without a personal or family history of liver or brain diseases and with normal liver enzyme levels. The study was conducted in accordance with the Declaration of Helsinki and approved by the Ethics Committee of the First Hospital of Jilin University (approval number: 2023-430).

#### Sample collection

Fasting blood samples from WD patients and healthy controls were collected in 6 mL EDTA anticoagulant tubes in the morning. The blood samples were centrifuged at 4,000 rpm for 5 min within 2 h to separate serum and plasma. Subsequently, the plasma and serum samples were collected and immediately stored at -80°C for subsequent proteomic analysis and ELISA verification.

### Proteomic analysis

#### Proteomic sample preparation

Plasma samples were taken out from -80°C and centrifuged (4°C, 12,000 g, 10 min). High-abundance proteins in plasma were depleted using the Pierce™ Top 14 Abundant Protein Depletion Spin Columns Kit (Thermo Scientific) according to the manufacturer’s instructions, and protein concentration was determined by BCA assay (Thermo Fisher). An equal amount of protein from each sample was subjected to enzymatic hydrolysis. Dithiothreitol was added to adjust the final concentration to 5 mM, and after reduction at 56°C for 30 min, iodoacetamide was added to adjust the final concentration to 11 mM. The samples were placed in ultrafiltration tubes and centrifuged (12,000 g, 20 min), followed by buffer exchange with 8 M urea and buffer for 3 times respectively. Trypsin was then added at a ratio of 1: 50 (protease: protein, m/m) for overnight digestion. Peptides were recovered by centrifugation at room temperature (12,000 g, 10 min) and then recovered once with ultrapure water, and the peptide solutions from the two times were combined.

### LTQ-orbitrap detection

After separation by an ultra-high performance liquid system, the peptides were injected into the NSI ion source for ionization and then analyzed by Qrbitrap Exploris™ 480 mass spectrometry. Firstly, the ion source voltage was 2.3 kV, and the FAIMS voltage was -45 and -70. High-resolution Qrbitrap was used to detect and analyze peptide parent ions and their secondary fragments, with the parameters as follows: the primary mass spectrometry scan range was 400-1,200 m/z, and the scan resolution was 60,000; the secondary mass spectrometry scan range was fixed at 110 m/z as the starting point, the scan resolution was 15,000, and TurboTMT was set to None. The data acquisition mode adopted the data-dependent scanning program.

### Proteomic data analysis

Proteomic data were generated using label-free quantitative proteomics. Retrieval was performed using Proteome Discoverer (v2.4.1.15). The database was Homo_sapiens_9606_PR_20201214.fasta, with a reverse database added to calculate the false discovery rate (FDR) caused by random matching and a contamination database added to eliminate the impact of contaminant proteins in the identification results. The enzymatic cleavage mode was set to Trypsin (Full); the number of missed cleavage sites was set to 2; the minimum length of peptides was set to 6 amino acid residues; the maximum number of modifications of peptides was set to 3; the mass error tolerance for primary parent ions was set to 10 ppm, and that for secondary fragment ions was 0.02 Da. Carbamidomethyl (C) was set as a fixed modification, and Oxidation (M), Acetyl (N-terminus), Met-loss (M), and Met-loss+acetyl (M) were set as variable modifications. The FDR for protein, peptide, and PSM identification was all set to 1%, and missing values were imputed by normal distribution (21). Proteins with >50% missing values in both groups were excluded, resulting in 1,404 proteins for downstream analysis. Pearson correlation, principal component analysis, and relative standard deviation were used to evaluate the repeatability of protein quantification.

### Differential expression analysis

Differentially expressed proteins (DEPs) in WD patients versus controls were identified using the limma package (R v4.0.3), with significance defined as |fold change (FC)| > 1.3 or |FC| < 0.769 and adjusted p < 0.05 (Benjamini-Hochberg correction). Visualization of DEPs was performed by generating heatmaps with the pheatmap package and volcano plots using ggplot2 in R.

### Drug target prediction

Known WD drugs and their corresponding targets were retrieved from the DrugBank database (https://go.drugbank.com/) using the query “Wilson Disease.” Overlaps between DrugBank targets and DEPs were identified using Venny 2.1, yielding a set of hub proteins for functional validation.

### Functional enrichment analysis

Gene Ontology (GO) and Kyoto Encyclopedia of Genes and Genomes (KEGG) pathway enrichment analyses were performed on DEPs using Gene Set Enrichment Analysis (GSEA) (https://www.gsea-msigdb.org/gsea/index.jsp). Analyses were conducted respectively on biological processes (BP), cellular components (CC), and molecular functions (MF) to understand the signaling pathways involved in the occurrence and development of diseases. Significance was defined as p < 0.05, with pathway visualization via ggplot2 and Cytoscape.

### ELISA validation

Serum samples from 10 WD patients and 10 controls were collected. Protein levels of Inter-alpha-trypsin inhibitor heavy chain 1 (ITIH1), Transthyretin (TTR), and CP were measured using human ELISA kits (Shanghai Keaibio, CB11750-Hu for ITIH1, CB11791-Hu for TTR, CB10305-Hu for CP) according to standard protocols. Protein concentrations were determined via a four-parameter logistic (4-PL) model in GraphPad Prism 9. Statistical comparisons between groups were performed using the two-tailed Student’s t-test, with significance set at p<0.01.

### Single-cell transcriptomics analysis

#### Data processing

scRNA-seq data was obtained from Gene Expression Omnibus (https://www.ncbi.nlm.nih.gov/, accession number: GSE254082). Hepatic mesenchymal cells from three WD patients and three hepatic hemangioma patients were subjected to scRNA-seq analysis. Raw FASTQ files were processed with Cell Ranger v3.1.0 to generate gene expression matrices, which were subsequently analyzed using the Seurat package (v4.1.0). Low-quality cells were filtered out by retaining cells with ≥300 detected genes and ≥10 unique molecular identifiers (UMIs). Data normalization was performed using the “NormalizeData” function with the “LogNormalize” method. Variable features were identified using “FindVariableFeatures” function to recognize the top 5,000 genes, followed by principal component analysis (PCA) with “RunPCA” function. Batch effects were corrected using the Harmony package (v0.1.0).

### Cell subset clustering and annotation

Cell clusters were identified using “FindClusters” functions (resolution = 0.5). Uniform manifold approximation and projection (UMAP) was applied for dimensionality reduction and visualization. Cell types were annotated based on canonical marker genes from the CellMarker database (http://117.50.127.228/CellMarker/index.html).

### Differentially expressed gene analysis

Differentially Expressed Gene (DEG) analysis between cell subsets was performed using “FindAllMarkers” with parameters: min.pct = 0.25, logfc.threshold = 0.25. Genes with adjusted p < 0.05 were considered differentially expressed.

### Pathway enrichment and GSVA analysis

GO/KEGG enrichment analysis of DEGs was performed as described above. Gene Set Variation Analysis (GSVA) was conducted using the msigdbr and gsva package in R, applying hallmark gene sets from MSigDB to assess pathway activity across cell subsets.

### Immune infiltration analysis

Immune cell composition was estimated using CIBERSORT (v1.03) package with the LM22 signature matrix, which quantifies 22 immune cell types from bulk transcriptomic data. Proportions of immune cells were compared between WD patients and controls using the Wilcoxon rank-sum test. Correlations between hub proteins and immune cell proportions were evaluated via Pearson’s correlation coefficient.

### Subtype-specific biomarker identification

#### LASSO regression for feature selection

To identify proteins distinguishing hepatic and brain WD subtypes, DEPs between the two subtypes were first identified. LASSO (Least Absolute Shrinkage and Selection Operator) regression was applied using the glmnet package in R to reduce dimensionality and avoid overfitting. The optimal regularization parameter (λ) was selected via 10-fold cross-validation, yielding 10 candidate proteins with non-zero coefficients.

### SVM-REF for protein ranking

Support Vector Machine with Recursive Feature Elimination (SVM-REF) was performed to rank the 10 candidate proteins by importance. The algorithm iteratively removed the least significant feature until the top 10 proteins were ranked based on their contribution to SVM classification accuracy.

### Intersection analysis

The top 10 proteins from LASSO and SVM-REF were intersected using Venny 2.1. Their diagnostic performance was evaluated via receiver operating characteristic (ROC) curve analysis, with area under the curve (AUC) calculated using the pROC package.

### Statistical analysis

Statistical analyses were performed in R (v4.2.1). The Wilcoxon rank-sum test was used to compare protein expression, cell proportions, and pathway enrichment scores between WD and control groups. A two-tailed Student’s t-test was used to compare ELISA results. A p-value < 0.05 was considered statistically significant.

## Results

### Differential protein analysis between disease and normal samples

**Figure 1:**
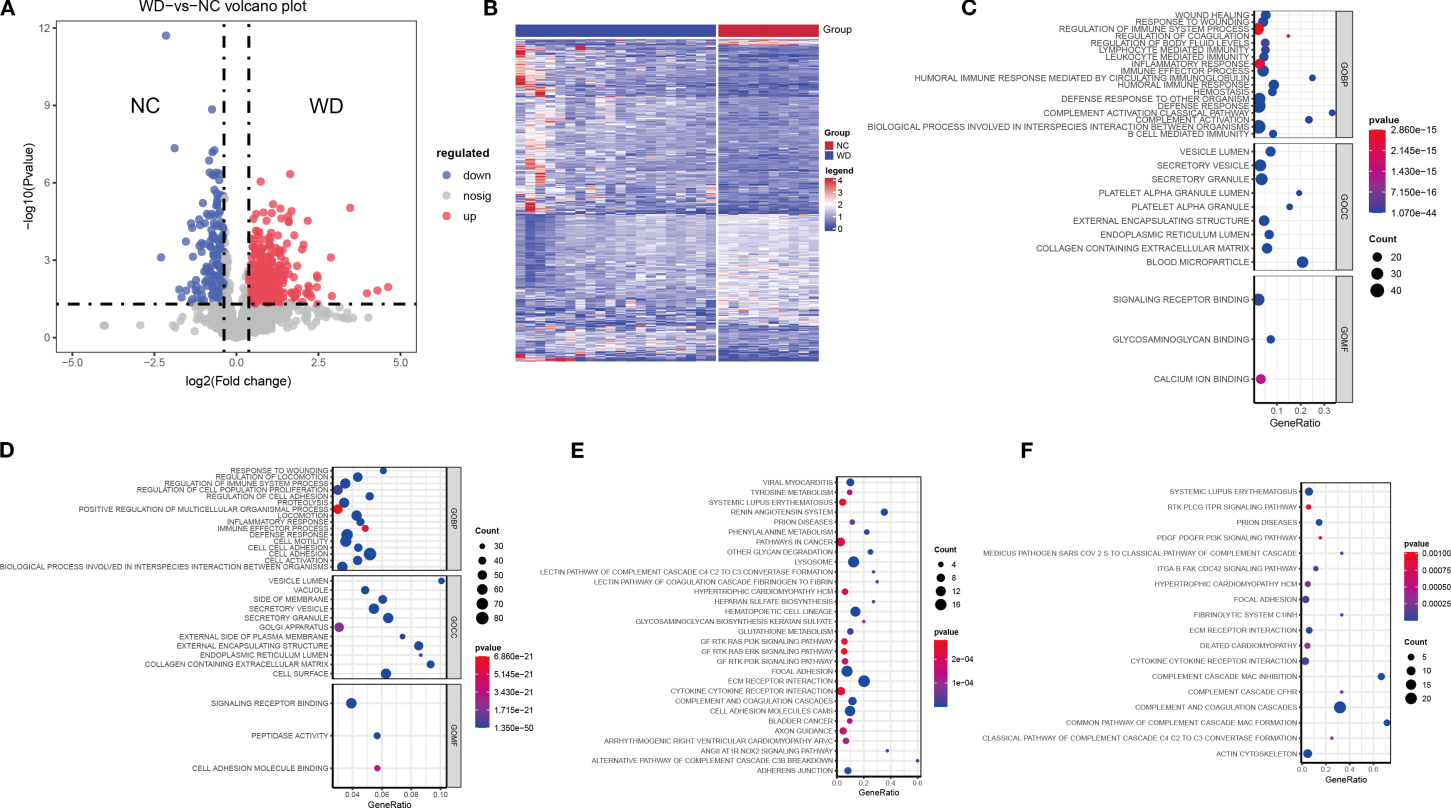
Differential protein expression and pathway enrichment between WD patients and controls. **(A)** Volcano plot showing differentially expressed proteins (DEPs) (adjusted p < 0.05, FC > 1.3 or < 0.769). Red points: upregulated proteins; blue points: downregulated proteins. **(B)** Heatmap of DEPs (n = 420), clustered by expression pattern across WD patients (n = 20) and controls (n = 10). **(C, D)** GO enrichment analysis of upregulated **(C)** and downregulated **(D)** DEPs, highlighting key biological processes (BP), cellular components (CC), and molecular functions (MF). **(E, F)** KEGG pathway enrichment of upregulated **(E)** and downregulated **(F)** DEPs. Dot size indicates protein count; color gradient reflects p-value significance.

Comparative proteomic analysis identified 420 DEPs between WD patients and health controls, including 266 upregulated and 154 downregulated proteins (**Figures 1A, B**; **Supplementary Table S1**). Functional enrichment analysis revealed significant activation of inflammatory pathways in upregulated DEPs. KEGG and GO analysis identified enrichment in “regulation of immune system process”, “inflammatory response” and “PI3K signaling pathway” (**Figures 1C, E**; **Supplementary Table S2**). Specifically, these processes encompassed “wound healing”, “regulation of coagulation”, “lymphocyte-mediated immunity”, “leukocyte-mediated immunity”, “immune effector process”, “humoral immune response mediated by circulating immunoglobulin”, and “defense response to other organism”. These pathways were closely associated with liver injury observed in WD. Downregulated DEPs were shown in **Figures 1D, F** (**Supplementary Table S3**), these proteins were significantly enriched in biological processes related to wound healing, immune responses, cell proliferation, and locomotion, as well as cellular components such as vesicles, membranes, and extracellular matrices. Enriched molecular functions include peptidase activity and cell adhesion molecule binding. Pathway analysis also revealed involvement in autoimmune diseases (e.g., Systemic Lupus Erythematosus), signaling pathways (e.g., RTK PLCG ITPR), and complement cascades.

### Differential analysis across WD subtypes

**Figure 2:**
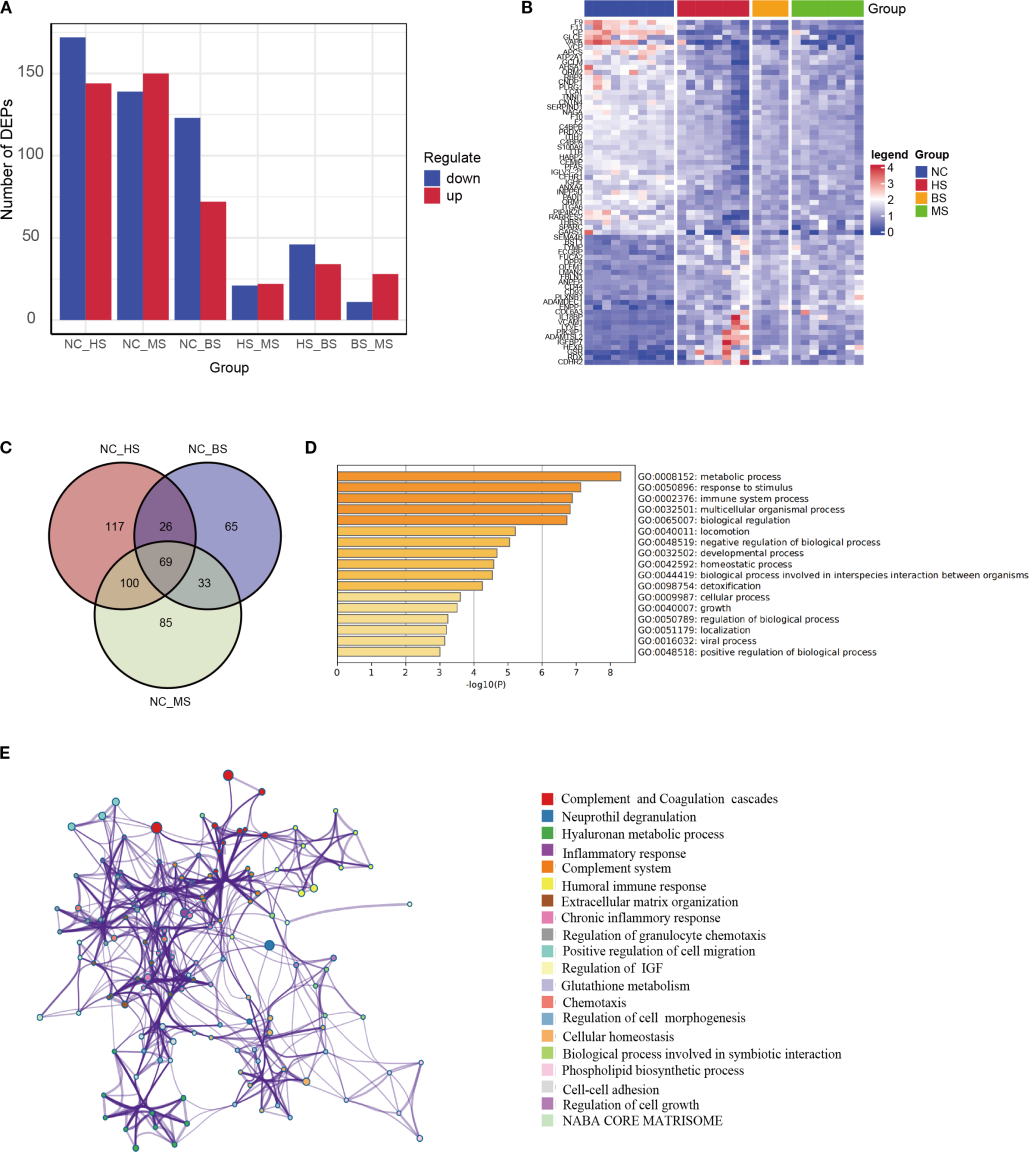
Subtype-specific protein expression and key pathway analysis. **(A)** Bar chart showing the number of DEPs in hepatic (HS), brain (BS), and mixed (MS) subtypes compared to controls (15), and between subtypes. **(B)** Heatmap of DEPs among different subtypes. **(C)** Venn diagram of overlapping DEPs across all subtypes vs. controls, yielding 69 key proteins. **(D, E)** GO functional enrichment **(D)** and pathway interaction network **(E)** of the 69 key proteins, annotated to inflammation, metabolism, and immune response.

We analyzed the DEPs across HS, BS, MS and health normal control (NC) group for the distinct DEP profiles in WD. The results showed that there was the highest variable dominance of up- or downregulated DEPs in the WD subtype groups (NC vs HS, NC vs BS, NC vs MS) (**Figures 2A, B**), supporting a specific molecular mechanism.

To identify core proteins driving WD pathogenesis, an intersection analysis was performed on DEPs from HS, BS, and MS versus NC, yielding 69 shared key proteins (**Figure 2C**, **Supplementary Table S4**). The functional enrichment analysis reveals that DEPs are significantly involved in metabolic processes, immune responses, and stimulus response, with additional enrichment in multicellular organismal processes, biological regulation, and development. Moderate enrichment is seen in locomotion, homeostasis, detoxification, growth, and viral processes (**Figures 2D, E**). These findings highlight the widespread biological impact, particularly in metabolism, immunity, and regulatory mechanisms.

### Drug target identification and hub protein analysis

**Figure 3:**
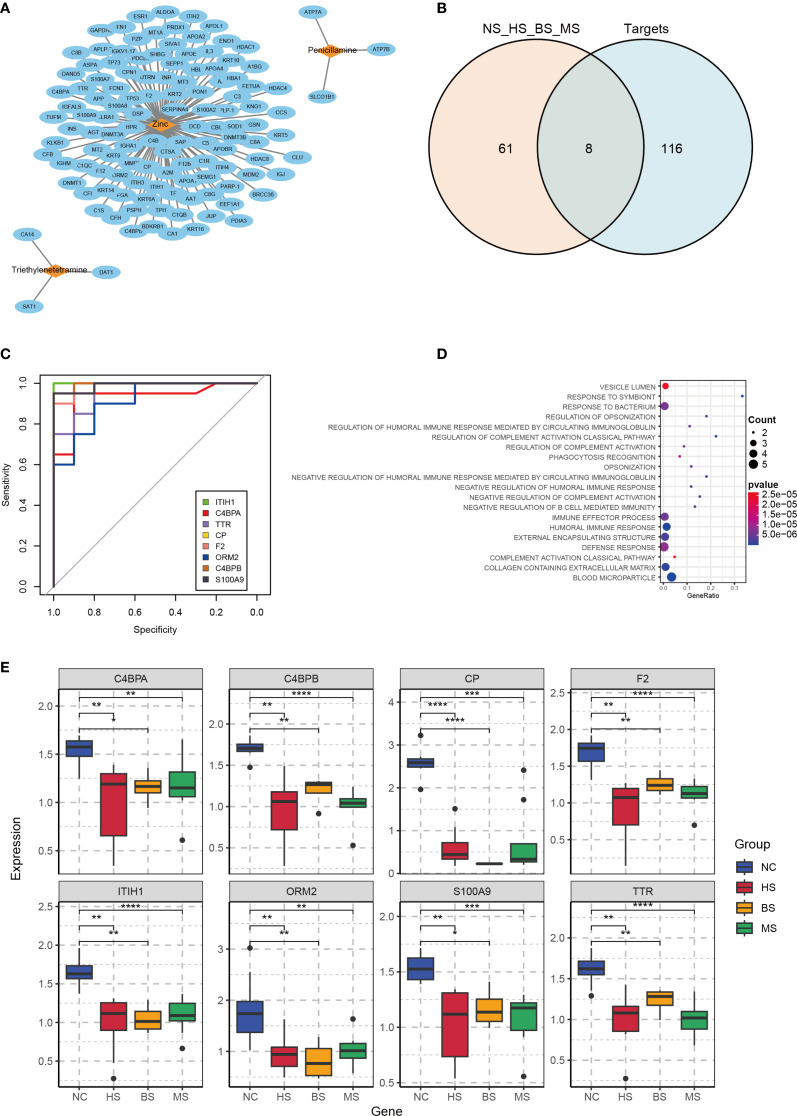
Drug target analysis and hub protein characterization. **(A)** Network diagram of WD therapeutic targets from DrugBank (nodes: drugs; edges: targets). **(B)** Venn diagram showing overlap between DrugBank targets and DEPs, identifying 8 hub proteins. **(C)** ROC curves for hub proteins, with AUC values indicating predictive accuracy. **(D)** KEGG pathway enrichment of hub proteins, highlighting immune and metabolic pathways. **(E)** Boxplots of hub protein expression in WD subtypes and controls (NC, control group; HS, hepatic subtype; BS, brain subtype; MS, mixed subtype; *p< 0.05, **p < 0.01, ***p < 0.001, ****p < 0.0001).

Retrieval from DrugBank identified three approved WD therapies: zinc, penicillamine, and triethylenetetramine, with known targets shown in **Figure 3A**. By intersecting the target proteins with DEPs, we identified eight hub proteins: ITIH1, Complement component 4 binding protein alpha (C4BPA), TTR, CP, Coagulation factor II (F2), Orosomucoid 2 (ORM2), Complement component 4 binding protein beta (C4BPB), and S100 calcium binding protein A9 (S100A9) (**Figure 3B**). To evaluate the ability of these eight proteins to discriminate between patients and healthy individuals, ROC curves were constructed. The results demonstrated that most of these proteins exhibited excellent diagnostic performance, with AUC values exceeding 0.9 (**Figure 3C**).

The hub proteins were found to be associated with pathways including the immune system, inflammatory response, and cellular metabolism. In the context of the immune system, these proteins play a role in generating antibodies to combat foreign pathogens or autoantigens. However, the toxic effects of copper may interfere with this process, leading to abnormal antibody production and compromised immune function. Additionally, in WD, copper deposition can trigger acute inflammatory responses, resulting in tissue damage and dysfunction. Specifically, copper-induced inflammation may activate pro-inflammatory signaling cascades, exacerbating hepatic and brain injury. Copper metabolic abnormalities in WD can disrupt the positive regulation of protein metabolic processes, leading to abnormalities in protein synthesis or degradation. This suggests that various metabolic pathways in WD patients may be abnormally activated, contributing to the disease’s pathophysiology (**Figure 3D**). Expression boxplots of these proteins across different groups showed that all hub proteins were downregulated in the disease group compared to controls, while there were no significant differences in expression among the hepatic, brain, and mixed subtypes (**Figure 3E**).

**Figure 4:**
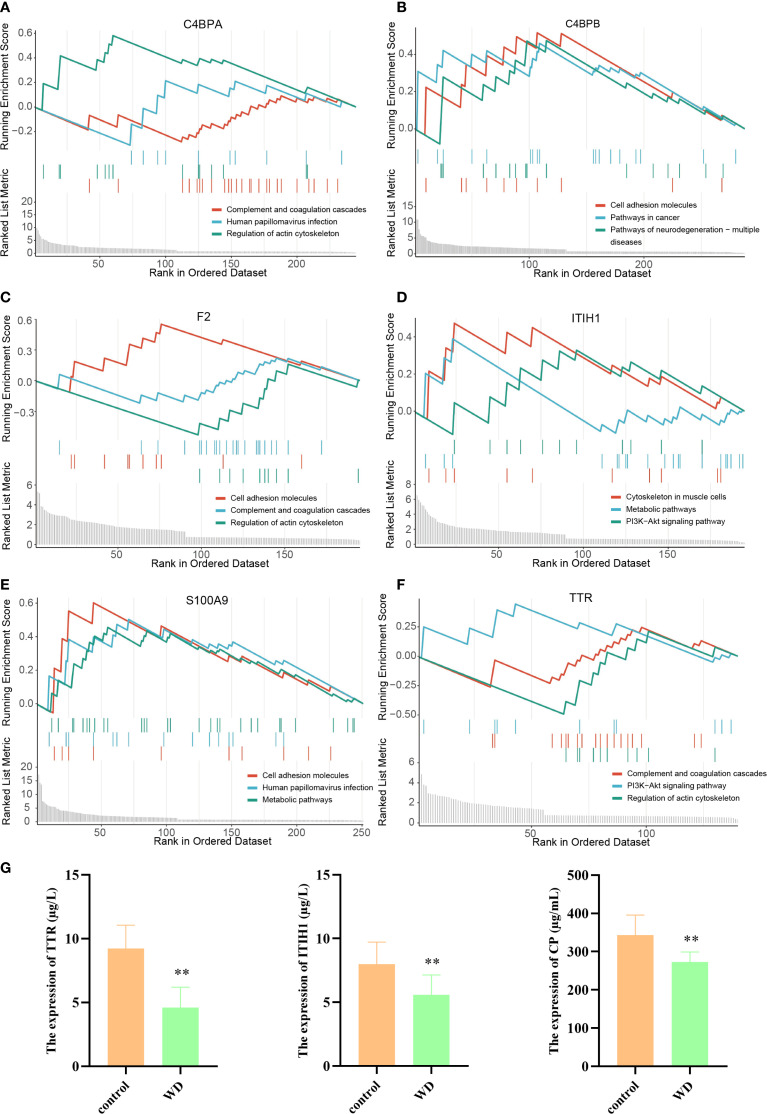
GSEA analysis of hub proteins in WD and ELISA validation. **(A–F)** Gene Set Variation Analysis (GSVA) plots showing regulation pathway of C4BPA, C4BPB, F2, ITIH1, S100A9, and TTR. **(G)** Target protein expression of WD patients and controls with ELISA (**p<0.01).

Subsequently, hub proteins were grouped by high and low expression to identify differentially regulated proteins, followed by GSEA to explore potential regulatory pathways. Results indicated that ITIH1 and TTR were significantly associated with the PI3K-Akt signaling pathway (**Figures 4A–F**). CP, a marker associated with copper metabolism, is closely related to WD and can serve as a biological indicator for WD detection (22, 23). Therefore, ELISA validation was performed for TTR, ITIH1, and CP. The levels of TTR, ITIH1, and CP in the serum of WD patients were significantly lower than those in the control group (**Figure 4G**), suggesting that these proteins are involved in the core pathological mechanisms of WD.

### Immune infiltration analysis in WD

**Figure 5:**
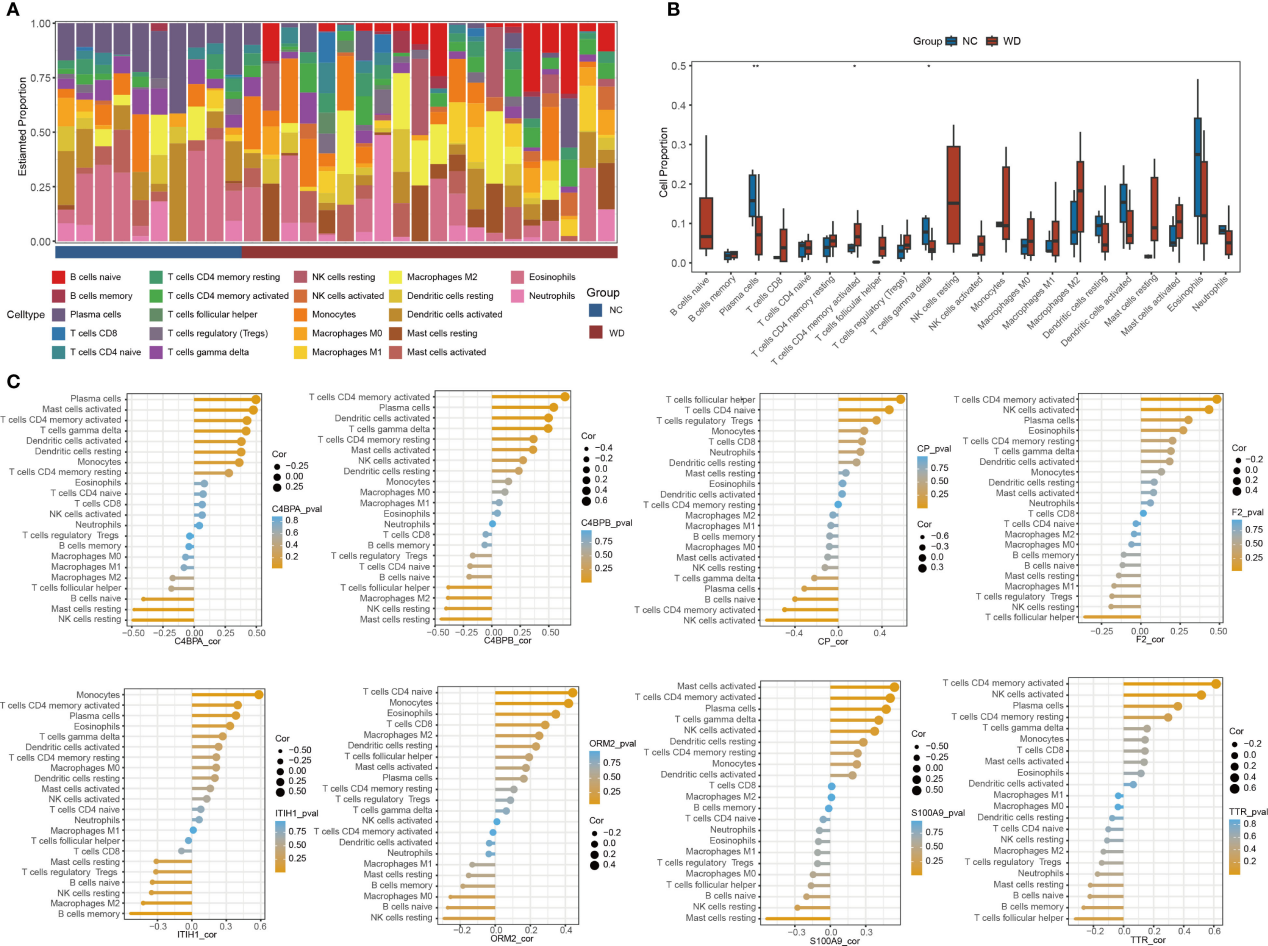
Immune cell infiltration analysis. **(A)** Stacked bar plot of immune cell proportions in WD patients and controls. **(B)** Boxplots comparing immune cells between groups (*p < 0.05, **p < 0.01). **(C)** Correlation between hub proteins and immune cell types.

CIBERSORT analysis was conducted to profile the distribution of 22 immune cell types across WD patient and control samples (**Figure 5A**). The results revealed significant differences in the proportions of plasma cells, CD4+ memory activated T cells, and γδ T cells between the disease and control groups (**Figure 5B**). Among then, compared with the NC group, the WD group showed a significant downregulation in plasma cells and γδ T cells (P < 0.01, P < 0.05), while a significant upregulation was observed in CD4+ memory activated T cells (P < 0.05). Correlation analysis further demonstrated associations between hub protein expression and immune cell composition (**Figure 5C**). These findings highlight immune cell dysregulation as a key feature of WD pathogenesis and suggest potential crosstalk between hub protein-mediated pathways and immune cell trafficking.

### Single-cell reveals the immune microenvironment of WD

**Figure 6:**
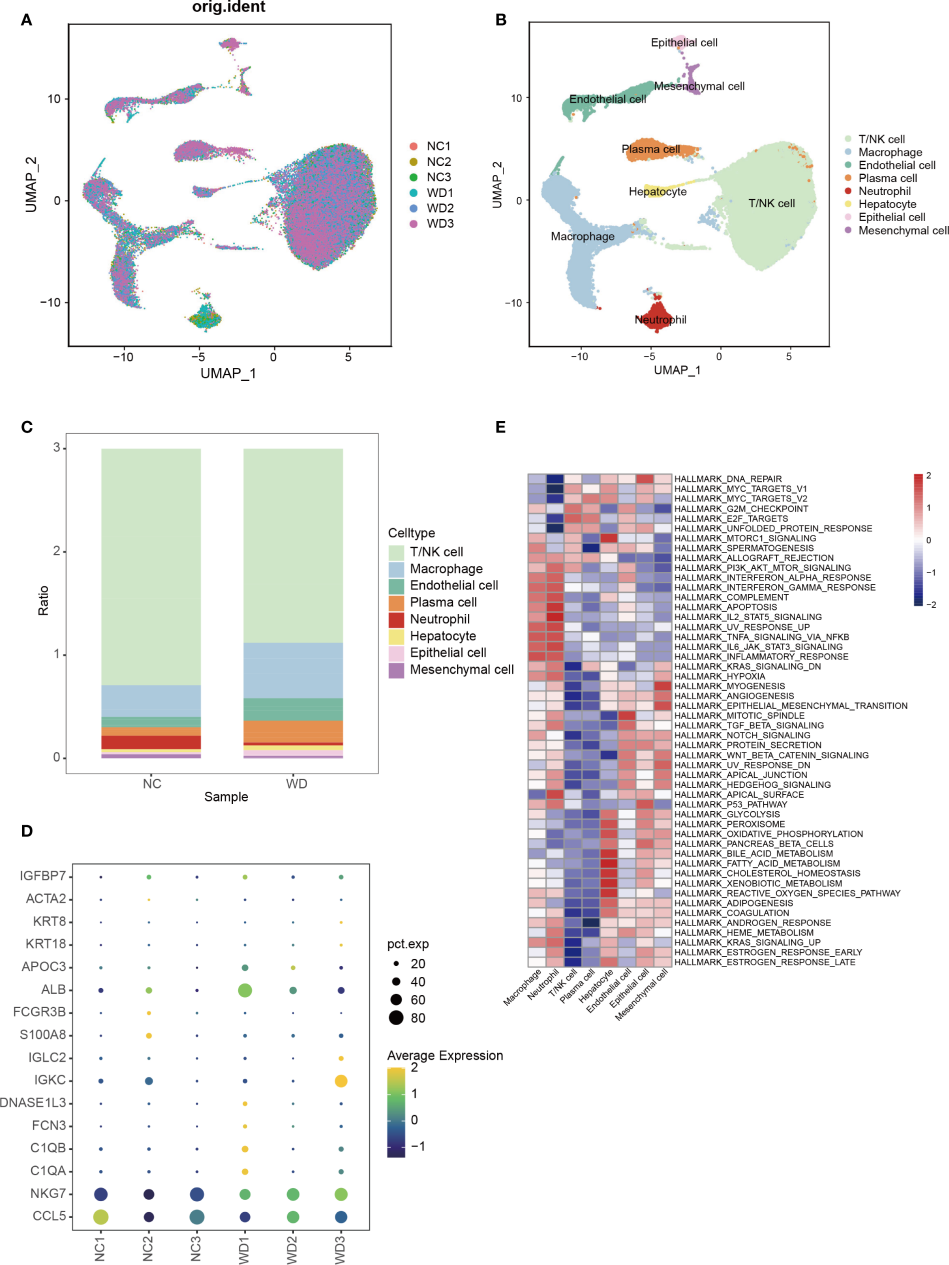
Single-cell transcriptomics of hepatic immune microenvironment. **(A)** UMAP plot showing cell distribution across WD patients (WD1-3) and controls (CTL1-3). **(B)** UMAP clustering of 8 cell types in WD patients. **(C)** Bar plot comparing immune cell proportions between WD patients and controls. **(D)** Bubble plot of marker protein expression between WD patients and controls. **(E)** GSVA enrichment of different cell clusters from WD patients.

Using the publicly available scRNA-seq dataset GSE254082 (3 WD patients vs. 3 liver hemangioma controls), we characterized hepatic immune cell heterogeneity. UMAP visualization identified eight distinct cell clusters, including hepatocytes, macrophages, T/NK cells, and endothelial cells, etc. (**Figures 6A, B**) WD samples showed a significant increase in macrophage proportion and reduction in T/NK cells compared to controls (**Figure 6C**). Marker protein analysis between WD patients and controls is shown in **Figure 6D**. GSVA further indicated enrichment of the PI3K-Akt-mTOR pathway in macrophages from WD patients (**Figure 6E**), linking macrophage activation to the pro-fibrotic and inflammatory phenotype observed in WD livers.

### Differential analysis between hepatic and brain subtypes

**Figure 7:**
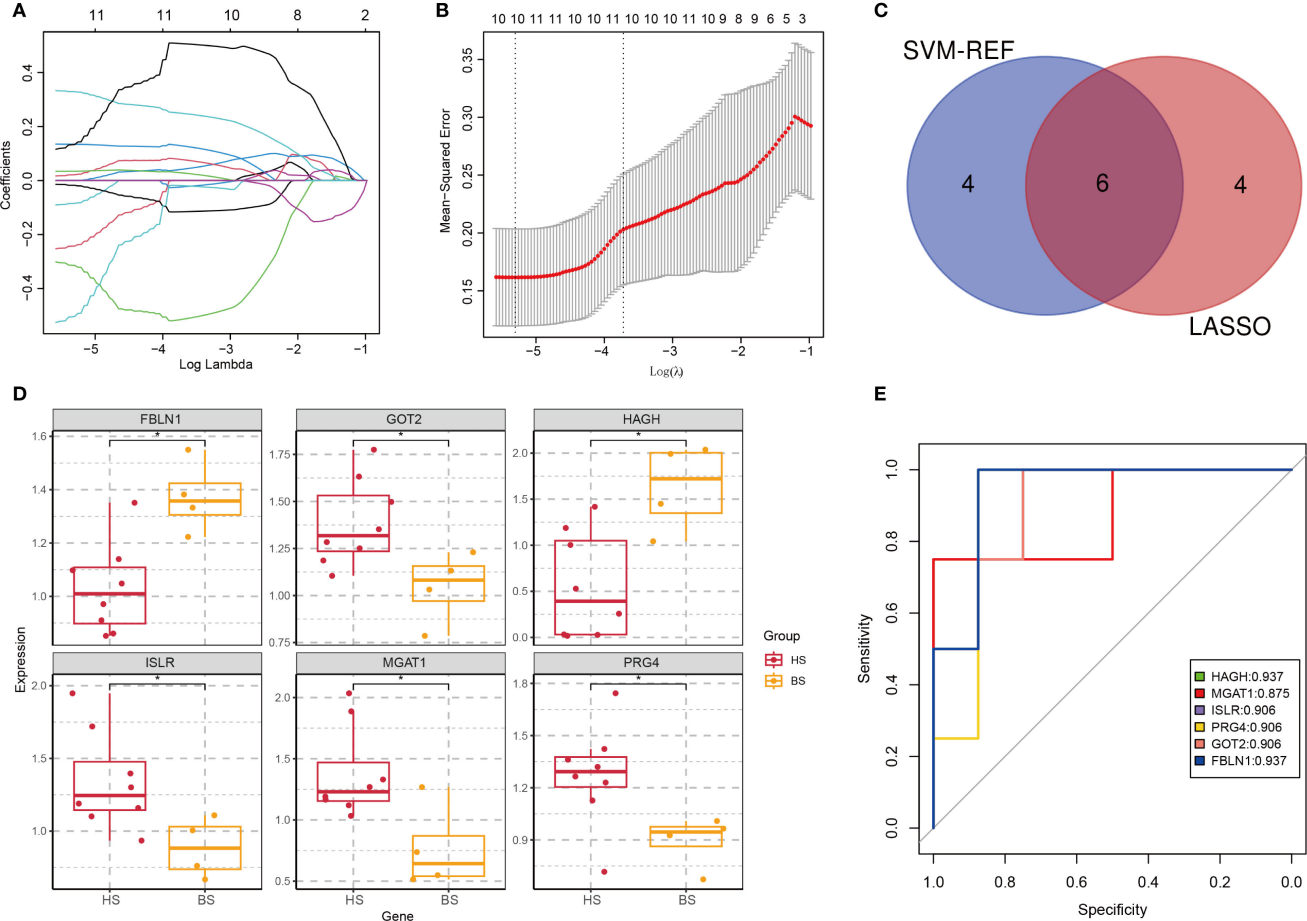
Identification of subtype-specific biomarkers. **(A)** Coefficient path plot. The horizontal axis represents the logarithm of the regularization parameter λ (lambda) in LASSO regression, and the vertical axis represents the coefficient values of each feature (variable). Each line in the plot represents the path of a feature’s coefficient as the regularization parameter λ changes. **(B)** Mean Squared Error (MSE) plot. The λ value that minimizes the MSE is selected as the optimal regularization parameter. **(C)** Venn diagram of top proteins from LASSO and SVM-REF. **(D)** Expression profiles of 6 biomarkers in hepatic (HS) and brain (BS) subtypes (*p < 0.05). **(E)** ROC curves evaluating protein diagnostic accuracy (AUC values indicated).

To identify subtype-specific biomarkers, we applied LASSO regression to 59 DEPs distinguishing hepatic and brain subtypes, yielding 10 candidate proteins (**Figures 7A, B**; **Supplementary Table S5**). SVM-REF further ranked these candidates, and intersection analysis identified six key proteins: Fibulin 1 (FBLN1), Glutamic-Oxaloacetic Transaminase 2 (GOT2), Hydroxyacylglutathione Hydrolase (HAGH), Insulin-like Growth Factor Binding Protein 3 Receptor (ISLR), Monoacylglycerol Acyltransferase 1 (MAGAT1), and Proteoglycan 4 (PRG4) (**Figure 7C**). Expression analysis showed FBLN1 and HAGH were upregulated in brain subtype, while GOT2, ISLR, MAGAT1, and PRG4 were elevated in hepatic subtype (**Figure 7D**). ROC curve analysis demonstrated robust diagnostic accuracy for all six proteins, with AUC values higher than 0.9 (**Figure 7E**). These findings provide a theoretical basis for the development of new therapeutic strategies and therapeutic targets.

## Discussion

WD is characterized by an exceptionally wide range of symptomatic variability and is recognized as a rare hereditary disorder with potentially fatal outcomes (24). Therefore, in-depth studies on the pathogenesis and potential therapeutic targets of WD are of great significance. This study integrated proteomics and single-cell transcriptomics to explore the molecular mechanisms of WD. Through comparative analysis of WD patients and healthy controls, 420 DEPs were identified. Proteomic analysis uncovered 420 DEPs linked to inflammation and metabolic dysfunction, with eight hub proteins (ITIH1, C4BPA, TTR, CP, F2, ORM2, C4BPB, S100A9) demonstrating robust diagnostic accuracy (AUC > 0.9). In addition, ELISA validation was performed for ITIH1 and TTR (which may be involved in the regulation of the PI3K-Akt signaling pathway) and CP (used for biological detection of WD), and the results showed a significant downregulation trend in ITIH1, TTR, and CP, providing new targets and evidence for the clinical diagnosis and treatment of WD. Since proteomics is based on the expression levels of entire cell populations and ignores the heterogeneity of the hepatic immune microenvironment, scRNA-seq technology, which enables high-throughput analysis at the single-cell level, fully preserves the diversity of individual cells in the data. Results from immune infiltration and single-cell transcriptomics analyses indicated that WD patients exhibit immune microenvironment imbalance, manifested as abnormal proportions of peripheral blood immune cells, activation of hepatic macrophages, and a reduction in neutrophils. More importantly, the PI3K-Akt signaling pathway was significantly enriched at multiple levels, suggesting that it plays a central role in disease progression. These findings collectively reveal the interactions between copper toxicity, immune dysregulation, and subtype-specific pathways, providing directions for precise treatment. Furthermore, we identified six key proteins (FBLN1, GOT2, HAGH, ISLR, MAGAT1, and PRG4) that can effectively distinguish between the hepatic and brain subtypes of WD. These findings collectively highlight the interplay between copper toxicity, immune dysregulation, and subtype-specific pathways, providing a roadmap for precision therapy.

In WD, copper toxicity disrupts metabolic processes and induces endoplasmic reticulum stress, which in turn leads to protein misfolding and defective secretion (25). These pathological changes activate innate immune responses and pro-inflammatory signaling pathways, ultimately resulting in tissue damage (26). Therefore, therapeutic strategies targeting the regulation of inflammatory responses or the restoration of metabolic homeostasis are expected to serve as effective supplements to traditional copper-lowering therapies (27). This study identified 8 key candidate molecules (ITIH1, C4BPA, TTR, CP, F2, ORM2, C4BPB, S100A9), whose expression was generally downregulated in WD patients, suggesting their potential as biomarkers for the early diagnosis of WD. These proteins play crucial roles in maintaining normal physiological functions of the body, particularly in immune and inflammatory responses (ITIH1, C4BPA, C4BPB, ORM2, S100A9), substance transport (TTR), metal metabolism and antioxidant defense (CP), and the coagulation system (F2) (28, 29). Furthermore, previous studies have shown that downregulation of ITIH1 can promote the progression of hepatocellular carcinoma by activating the PI3K/AKT signaling pathway (30); TTR reduces the occurrence of diabetic retinopathy by regulating the VEGFA/PI3K/AKT axis (31). Meanwhile, studies have reported that the PI3K-Akt pathway is closely associated with WD. For example, Zhang et al. found that activating the PI3K-AKT signaling pathway can alleviate cognitive impairment in WD (32). Notably, no studies have yet linked ITIH1 and TTR to the PI3K-Akt pathway in WD, while this study is the first to suggest that ITIH1 and TTR may be involved in the regulation of the PI3K-Akt signaling pathway in WD. These findings provide an important molecular biological basis for elucidating the pathogenesis of WD and offer new insights into exploring potential diagnostic markers and therapeutic targets.

To reveal the diversity and dynamic changes of different immune cells, we performed immune infiltration analysis and single-cell transcriptome data analysis. Immune infiltration analysis showed that the composition of immune cells in WD patients was altered, including an increase in activated CD4+ memory T cells and a decrease in plasma cells and γδ T cells. These changes indicate that WD is undergoing a shift toward chronic immune activation and autoimmunity, and this immune deficiency mediates the development of liver cirrhosis, which is associated with abnormalities in various innate and adaptive immune components (33). Single-cell analysis revealed an imbalance in the immune microenvironment of WD patients, characterized by a reduction in T/NK cells and neutrophils and an increase in macrophages. Previous studies have indicated that immune cells play a crucial role in the pathogenesis of WD-related liver injury (34, 35). For example, Li et al. reported that an imbalance in immune cell populations can exacerbate tissue damage under copper-overload conditions (36). Macrophages, in particular, are known to be involved in the inflammatory response triggered by copper toxicity (37). Interestingly, this study found that the PI3K-Akt-mTOR pathway is enriched in macrophages of WD patients, further confirming the critical role of this pathway in WD. Genes in the PI3K-Akt pathway have been implicated in hepatocyte apoptosis and fibrosis triggered by copper overload (38, 39), suggesting its pro-inflammatory and pro-fibrotic effects in WD liver pathology. This pathway activation can lead to a pro-fibrotic and inflammatory phenotype, as observed in WD livers. This finding is consistent with previous studies on Gandoufumu Decoction (GDFMD), which alleviates copper-induced liver injury by activating the PI3K-Akt-mTOR pathway to inhibit excessive autophagy (40). The results of proteomic and single-cell analyses in this study further confirm that the PI3K-Akt pathway plays an important role in the pathogenesis of WD, suggesting that interventions targeting this pathway may be beneficial for the treatment of WD.

To further explore subtype-specific biomarkers that can facilitate precision diagnosis and treatment, this study specifically screened and identified six candidate proteins (FBLN1, GOT2, HAGH, ISLR, MAGAT1, and PRG4) that can effectively distinguish between the hepatic and brain subtypes of WD. Among them, FBLN1 and HAGH are upregulated in the brain subtype, while GOT2, ISLR, MAGAT1, and PRG4 show increased expression in the hepatic subtype. These unique expression patterns may reflect differences in tissue-specific pathogenic mechanisms. FBLN1, an important extracellular matrix protein, is significantly upregulated in Alzheimer’s disease and brain metastases of non-small cell lung cancer (41, 42). GOT2 is involved in key metabolic pathways that could maintain liver cell homeostasis (43). HAGH is a neurology-related protein that is positively correlated with muscle strength. During childhood obesity, it has been found that muscle strength can maintain better brain health (44). ISLR has been implicated in hepatic stellate cell activation and fibrosis progression (45). MAGAT1 may be essential for liver regeneration after ischemia/reperfusion injury (46). PRG4, though originally linked to joint lubrication, has emerging roles in hepatic protection and anti-inflammatory responses (47). In the realm of precision medicine, biomarkers are essential for accurate diagnosis and tailored treatment. Recently, research on hepatocellular carcinomas and cholangiocarcinoma also demonstrated that subtype-specific biomarkers can enhance the accuracy of disease diagnosis and guide personalized therapeutic strategies (48, 49). Our identified biomarkers, with their high diagnostic accuracy (AUC > 0.9), could enhance current WD diagnostic strategies and enable the development of subtype-directed treatments. Targeting the associated pathways in distinct WD subtypes may lead to more effective, individualized therapeutic approaches.

However, this study has certain limitations. The sample size was relatively small, which may affect the representativeness of the results and limit their generalization. Moreover, while we have identified potential biomarkers and pathways related to WD, the in-depth functional validation of these biomarkers and the exploration of the precise molecular mechanisms underlying the pathways are still lacking. Future studies should aim to recruit larger sample cohorts and conduct more in-depth mechanistic investigations to address these limitations and further advance our understanding of WD. Moreover, exploring the interplay between the immune system, copper metabolism, and other organ systems in WD will provide a more comprehensive understanding of the disease.

## Conclusion

This study comprehensively integrates proteomic analysis and single-cell transcriptomic analysis. Eight proteins with high diagnostic accuracy were identified, among which ITIH1 and TTR may affect WD by regulating the PI3K-Akt pathway. In addition, we also explored six key proteins that can effectively distinguish between hepatic and brain subtypes of WD. Therefore, the findings of this study contribute to a deeper understanding of the pathogenesis of WD, aiming to provide new strategies for the clinical treatment of WD.

## Data Availability

The datasets presented in this study can be found in online repositories. The names of the repository/repositories and accession number(s) can be found in the article/**Supplementary Material**.
